# Supplementary material for: The prone position in COVID-19 impacts the thickness of peripapillary retinal nerve fiber layers and macular ganglion cell layers
Source: PLoS One. 2024 May 2;19(5):e0300621. doi: 10.1371/journal.pone.0300621 (PMC11065264; doi:10.1371/journal.pone.0300621)
Supplement: S1 Table — mGCIPL: Macular ganglion cell+inner plexiform layer, Av: Average, Min: Minimum, NS: Nasal-superior, NI: Nasal-inferior, S: Superior, I: Inferior, TS: Temporal-superior, TI: Temporal-inferior, SD: Standard deviation, CI: Confidence interval, Significant differences are shown in bold. (DOCX) [file pone.0300621.s001.docx]

**Table 1 : Comparison of m-GCIPL in None-prone group in different times**

| **m-GCILP thickness** | **None-prone group** (n=23) | | | p-value of 3 times | **Mean differences between months** | | | **95% CI** | | |
| --- | --- | --- | --- | --- | --- | --- | --- | --- | --- | --- |
|  | **1 mo** Mean (SD) | **3 mo** Mean (SD) | **6 mo** Mean (SD) |  |  |  |  |  |  |  |
|  |  |  |  |  | 1-3 | 3-6 | 1-6 | 1-3 | 3-6 | 1-6 |
| Av (µm) | 82.74 (5.63) | 83.13 (5.17) | 82.87 (5.18) | 0.122 | -0.39 | 0.26 | -0.13 | -0.85 to 0.06 | -0.15 to 0.67 | -0.73 to 0.46 |
| Min (µm) | 79.65 (5.20) | 80.13 (4.95) | 79.96 (4.82) | 0.132 | -0.48 | 0.17 | -0.30 | -1.02 to 0.06 | -0.47 to 0.82 | -0.94 to 0.34 |
| NS (µm) | 84.96 (6.33) | 85.13 (6.34) | 85.35 (6.05) | 0.506 | -0.17 | -0.22 | -0.39 | -1.16 to 0.81 | -0.85 to 0.41 | -1.32 to 0.54 |
| NI (µm) | 83.26 (5.15) | 84.0 (5.41) | 84.09 (5.38) | **0.009** | **-0.74** | -0.09 | -0.83 | -**1.31 to -0.17** | -0.72 to 0.54 | -1.74 to 0.09 |
| S (µm) | 83.00 (6.99) | 83.26 (6.69) | 83.35 (6.34) | 0.453 | -0.26 | -0.09 | -0.35 | -0.96 to 0.43 | -0.81 to 0.64 | -1.14 to 0.44 |
| I (µm) | 80.57 (4.60) | 81.04 (4.03) | 80.48 (3.96) | 0.156 | -0.48 | 0.57 | 0.09 | -1.43 to 0.47 | -0.12 to 1.25 | -0.66 to 0.83 |
| TS (µm) | 81.70 (7.00) | 82.00 (6.29) | 81.87 (6.28) | 0.663 | -0.30 | 0.13 | -0.17 | -1.14 to 0.53 | -0.60 to 0.87 | -1.19 to 0.84 |
| TI (µm) | 82.87 (6.33) | 83.39 (5.43) | 83.09 (5.52) | 0.265 | -0.52 | 0.30 | -0.22 | -1.41 to 0.37 | -0.40 to 1.00 | -1.08 to 0.64 |

mGCIPL: macular ganglion cell+inner plexiform layer, Av: average, Min: minimum, NS: nasal-superior, NI: nasal-inferior, S: superior, I: inferior, TS: temporal-superior, TI: temporal-inferior
SD: standard deviation, CI: confidence interval, Significant differences are shown in bold.
