# Supplementary material for: The prone position in COVID-19 impacts the thickness of peripapillary retinal nerve fiber layers and macular ganglion cell layers
Source: PLoS One. 2024 May 2;19(5):e0300621. doi: 10.1371/journal.pone.0300621 (PMC11065264; doi:10.1371/journal.pone.0300621)
Supplement: S2 Table — None-prone: None-prone group, Prone: Prone group, Control: Control group, pRNFL: Peripapillary RNFL, Av: Average, S: Superior, N: Nasal, I: Inferior, T: Temporal, SD: Standard deviation, CI: Confidence interval, Significant differences are shown in bold. (DOCX) [file pone.0300621.s002.docx]

**Table 2 : Comparison of p-RNFL thickness in None-prone group, Prone group, and Control group at 3 and 6 month**

| **p-RNFL thickness** | **Mean** (SD) | | | p-value of 3 groups | **Mean differences between group** | | | **95% CI** | | |
| --- | --- | --- | --- | --- | --- | --- | --- | --- | --- | --- |
|  | **None-prone** (n=23) | **Prone** (n=15) | **Control** (n=23) |  |  |  |  |  |  |  |
|  |  |  |  |  | None-prone and Prone | None-prone and Control | Prone and Control | None-prone and Prone | None-prone and Control | Prone and Control |
| **1 month** |  |  |  |  |  |  |  |  |  |  |
| **Av. (µm)** | 103.39 (8.46) | - | 100.48 (7.60) | 0.226 | - | -2.913 | - | - | -7.69 to 1.87 | - |
| **S (µm)** | 131.96 (12.88) | - | 127.48 (15.31) | 0.289 | - | -4.478 | - | - | -12.89 to 3.93 | - |
| **N (µm)** | 75.61 (12.66) | - | 73.26 (10.74) | 0.501 | - | -2.348 | - | - | -9.32 to 4.63 | - |
| **I (µm)** | 134.22 (16.84) | - | 130.35 (16.86) | 0.440 | - | -3.870 | - | - | -13.88 to 6.15 | - |
| **T (µm)** | 73.00 (12.17) | - | 70.52 (8.04) | 0.420 | - | -2.478 | - | - | -8.61 to 3.65 | - |
| **3 months** |  |  |  |  |  |  |  |  |  |  |
| **Av. (µm)** | 102.30 (9.52) | 98.20 (8.74) | 100.48 (7.60) | 0.364 | 4.10 | 1.83 | -2.28 | -2.97 to 11.18 | -4.46 to 8.11 | -9.35 to 4.80 |
| **S (µm)** | 131.78 (12.90) | 120.80 (19.21) | 127.48 (15.31) | 0.112 | 10.98 | 4.30 | -6.68 | -1.72 to 23.69 | -6.98 to 15.59 | -19.38 to 6.02 |
| **N (µm)** | 73.39 (12.81) | 75.67 (10.59) | 73.26 (10.74) | 0.792 | -2.28 | 0.13 | 2.41 | -11.71to 7.16 | -8.26 to 8.52 | -7.03 to 8.52 |
| **I (µm)** | 131.70 (15.55) | 124.33 (18.00) | 130.35 (16.86) | 0.393 | 7.36 | 1.35 | -6.01 | -6.27to 21.00 | -10.77 to 13.46 | -19.65 to 7.62 |
| **T (µm)** | 72.43 (11.13) | 72.47 (8.51) | 70.52 (8.04) | 0.743 | -0.03 | 1.91 | 1.95 | -7.75 to 7.69 | -4.94 to 8.77 | -5.77 to 9.66 |
| **6 months** |  |  |  |  |  |  |  |  |  |  |
| **Av. (µm)** | 102.78 (8.21) | 96.40 (7.72) | 100.48 (7.60) | 0.058 | 6.38 | 2.30 | -4.08 | -0.05 to 12.82 | -3.41 to 8.02 | -10.51 to 2.36 |
| **S (µm)** | 131.61 (12.08) | 118.87 (18.21) | 127.48 (15.31) | **0.043** | **12.74** | 4.13 | -8.61 | **0.49 to 25.00** | -6.76 to 15.02 | -20.87 to 3.64 |
| **N (µm)** | 74.74 (12.90) | 74.20 (8.41) | 73.26 (10.74) | 0.902 | 0.54 | 1.48 | 0.94 | -8.57 to 9.65 | -6.62 to 9.57 | -8.17 to 9.65 |
| **I (µm)** | 132.35 (13.92) | 122.13 (17.19) | 130.35 (16.86) | 0.146 | 10.21 | 2.00 | -8.21 | -2.79 to 23.22 | -9.55 to 13.55 | -21.22 to 4.79 |
| **T (µm)** | 72.78 (11.56) | 70.67 (9.48) | 70.52 (8.04) | 0.699 | 2.12 | 2.26 | 0.15 | -5.94 to 10.17 | -4.89 to 9.42 | -7.91 to 8.20 |

None-prone: None-prone group, Prone: Prone group, Control: Control group, pRNFL: peripapillary RNFL, Av: average, S: superior, N: nasal, I: inferior, T: temporal
SD: standard deviation, CI: confidence interval, Significant differences are shown in bold.
