# Supplementary material for: The prone position in COVID-19 impacts the thickness of peripapillary retinal nerve fiber layers and macular ganglion cell layers
Source: PLoS One. 2024 May 2;19(5):e0300621. doi: 10.1371/journal.pone.0300621 (PMC11065264; doi:10.1371/journal.pone.0300621)
Supplement: S3 Table — None-prone: None-prone group, Prone: Prone group, Control: Control group, mGCIPL: Macular ganglion cell+inner plexiform layer, Av: Average, Min: Minimum, NS: Nasal-superior, NI: Nasal-inferior, S: Superior, I: Inferior, TS: Temporal-superior, TI: Temporal-inferior, SD: Standard deviation, CI: Confidence interval, Significant differences are shown in bold. (DOCX) [file pone.0300621.s003.docx]

**Table 3 : Comparison of m-GCIPL in None-prone group, Prone group, and Control group at 3 and 6 month**

| **m-GCILP thickness** | **Mean** (SD) | | | p-value of 3 groups | **Mean differences between group** | | | **95% CI** | | |
| --- | --- | --- | --- | --- | --- | --- | --- | --- | --- | --- |
|  | **None-prone** (n=23) | **Prone** (n=15) | **Control**(n=23) |  |  |  |  |  |  |  |
|  |  |  |  |  | None-prone and Prone | None-prone and Control | Prone and Control | None-prone and Prone | None-prone and Control | Prone and Control |
| **1 month** |  |  |  |  |  |  |  |  |  |  |
| Av (µm) | 82.74 (5.63) | - | 84.91 (4.24) | 0.146 | - | -2.17 | - | - | -0.79 to 5.14 | - |
| Min (µm) | 79.65 (5.20) | - | 82.13 (4.39) | 0.088 | - | -2.48 | - | - | -0.38 to 5.34 | - |
| NS (µm) | 84.96 (6.33) | - | 87.09 (4.38) | 0.191 | - | -2.13 | - | - | -1.10 to 5.36 | - |
| NI (µm) | 83.26 (5.15) | - | 86.04 (4.89) | 0.067 | - | -2.78 | - | - | -0.20 to 5.77 | - |
| S (µm) | 83.00 (6.99) | - | 85.43 (4.02) | 0.155 | - | -2.44 | - | - | -0.95 to 5.82 | - |
| I (µm) | 80.57 (4.60) | - | 83.87 (5.43) | **0.031** | - | -3.30 | - | - | 0.31 to 6.30 | - |
| TS (µm) | 81.70 (7.00) | - | 83.13 (5.05) | 0.429 | - | -1.44 | - | - | -2.19 to 5.06 | - |
| TI (µm) | 82.87 (6.33) | - | 84.35 (4.65) | 0.372 | - | -1.48 | - | - | -1.82 to 4.78 | - |
| **3 months** |  |  |  |  |  |  |  |  |  |  |
| Av (µm) | 83.13 (5.17) | 83.47 (3.18) | 84.91 (4.24) | 0.363 | -0.336 | -1.783 | -1.446 | -3.94 to 3.27 | -4.99 to 1.42 | -5.05 to 2.16 |
| Min (µm) | 80.13 (4.95) | 79.73 (3.71) | 82.13 (4.39) | 0.190 | 0.397 | -2.000 | -2.397 | -3.26 to 4.05 | -5.25 to 1.25 | -6.05 to 1.26 |
| NS (µm) | 85.13 (6.34) | 85.93 (5.44) | 87.09 (4.38) | 0.478 | -0.803 | -1.957 | -1.154 | -5.26 to 3.65 | -5.92 to 2.00 | -5.61 to 3.30 |
| NI (µm) | 84.0 (5.41) | 84.20 (4.89) | 86.04 (4.89) | 0.349 | -0.200 | -2.043 | -1.843 | -4.37 to 3.97 | -5.75 to 1.66 | -6.01to 2.32 |
| S (µm) | 83.26 (6.69) | 83.20 (4.16) | 85.43 (4.02) | 0.289 | 0.061 | -2.174 | -2.235 | -4.21 to 4.33 | -5.97 to 1.62 | -6.51 to 2.04 |
| I (µm) | 81.04 (4.03) | 82.00 (3.84) | 83.87 (5.43) | 0.114 | -0.957 | -2.826 | -1.870 | -4.70 to 2.78 | -6.15 to 0.50 | -5.61 to 1.87 |
| TS (µm) | 82.00 (6.29) | 83.97 (5.09) | 83.13 (5.05) | 0.754 | -1.067 | -1.130 | -0.064 | -5.62 to 3.48 | -5.17 to 2.91 | -4.61 to 4.49 |
| TI (µm) | 83.39 (5.43) | 83.13 (4.45) | 84.35 (4.65) | 0.710 | 0.258 | -0.957 | -1.214 | -3.77 to 4.28 | -4.53 to 2.62 | -5.24 to 2.81 |
| **6 months** |  |  |  |  |  |  |  |  |  |  |
| Av (µm) | 82.87 (5.18) | 83.53 (3.58) | 84.91 (4.24) | 0.300 | -0.664 | -2.043 | -1.380 | -4.33 to 3.01 | -5.30 to 1.22 | -5.05 to 2.29 |
| Min (µm) | 79.96 (4.82) | 79.80 (4.02) | 82.13 (4.39) | 0.176 | 0.157 | -2.174 | -2.330 | -3.51 to 3.82 | -5.43 to 1.08 | -5.99 to 1.33 |
| NS (µm) | 85.35 (6.05) | 86.00 (5.72) | 87.09 (4.38) | 0.548 | -0.652 | -1.739 | -1.087 | -5.06 to 3.76 | -5.66 to 2.18 | -5.50 to 3.32 |
| NI (µm) | 84.09 (5.38) | 84.00 (4.81) | 86.04 (4.89) | 0.337 | 0.087 | -1.957 | -2.043 | -4.06 to 4.23 | -5.64 to 1.72 | -6.19 to 2.10 |
| S (µm) | 83.35 (6.34) | 83.20 (4.78) | 85.43 (4.02) | 0.300 | 0.148 | -2.087 | -2.235 | -4.09 to 4.39 | -5.86 to 1.68 | -6.48 to 2.01 |
| I (µm) | 80.48 (3.96) | 82.33 (3.94) | 83.87 (5.43) | **0.049** | -1.855 | **-3.391** | -1.536 | -5.59 to 1.88 | **-6.71 to -0.07** | -5.28 to 2.20 |
| TS (µm) | 81.87 (6.28) | 82.60 (4.95) | 83.13 (5.05) | 0.741 | -0.730 | -1.261 | -0.530 | -5.25 to 3.79 | -5.28 to 2.76 | -5.05 to 3.99 |
| TI (µm) | 83.09 (5.52) | 82.20 (2.86) | 84.35 (4.65) | 0.367 | 0.887 | -1.261 | -2.148 | -2.93 to 4.70 | -4.65 to 2.13 | -5.96 to 1.66 |

None-prone: None-prone group, Prone: Prone group, Control: Control group,
mGCIPL: macular ganglion cell+inner plexiform layer, Av: average, Min: minimum, NS: nasal-superior, NI: nasal-inferior, S: superior, I: inferior, TS: temporal-superior, TI: temporal-inferior
SD: standard deviation, CI: confidence interval, Significant differences are shown in bold.
