# Supplementary material for: The prone position in COVID-19 impacts the thickness of peripapillary retinal nerve fiber layers and macular ganglion cell layers
Source: PLoS One. 2024 May 2;19(5):e0300621. doi: 10.1371/journal.pone.0300621 (PMC11065264; doi:10.1371/journal.pone.0300621)
Supplement: S4 Table — None-prone: None-prone group, Prone: Prone group, VFI: Visual field index, MD: Mean deviation, PSD: Pattern standard deviation, SD: Standard deviation, Significant differences are shown in bold. (DOCX) [file pone.0300621.s004.docx]

**Table 4 : Comparison of visual field parameters in None-prone group and Prone group at 1, 3 and 6 month**

| **HVF 24-2**  **parameters** | **None prone group** | | | | **Prone group** | | |
| --- | --- | --- | --- | --- | --- | --- | --- |
|  | **1 mo**  Mean (SD) | **3 mo**  Mean (SD) | **6 mo**  Mean (SD) | p-value | **3 mo**  Mean (SD) | **6 mo**  Mean (SD) | p-value |
| **VFI (%)** | 97.87 (1.94) | 98.43 (1.75) | 98.22 (2.07) | 0.349 | 93.79  (6.69) | 95.93  (4.94) | 0.179 |
| **MD (dB)** | -1.70  (1.91) | -1.29  (1.97) | -1.42  (1.61) | 0.592 | -3.51  (3.47) | -2.52  (2.21) | 0.252 |
| **PSD (dB)** | 1.98  (0.60) | 1.80  (0.59) | 1.93  (0.78) | 0.413 | 4.10  (3.04) | 3.26  (2.81) | 0.130 |
| **Pattern** | No defect | No defect | No defect | - | No defect | No defect | - |

None-prone: None-prone group, Prone: Prone group, VFI : visual field index , MD : mean deviation ,PSD : pattern standard deviation, SD: standard deviation, Significant differences are shown in bold.
