# Supplementary material for: The prone position in COVID-19 impacts the thickness of peripapillary retinal nerve fiber layers and macular ganglion cell layers
Source: PLoS One. 2024 May 2;19(5):e0300621. doi: 10.1371/journal.pone.0300621 (PMC11065264; doi:10.1371/journal.pone.0300621)
Supplement: Attachment — Submitted filename: PLOS ONE Feb 2024.docx [file pone.0300621.s005.docx]

**PLOS ONE	Feb 2024**

**Reviewer 1**

The manuscript is well written and the interpreation of the findings sound. I just have 2 comments

1- The manuscript would have benefited from performing OCT angiography to study the ischemic changes if present. This could be added to the limitations.

2- I cant find the results of the visual field testing.

**Reviewer 2**

The research question is discussing a very novel point

The methodology and data analysis are presented in a very good manner

The discussion section requires revision and editing for data to be presented in a more organized and explanatory style

**Editor**

Since Ischemic Optic Neuropathy is the main presumed pathogenesis , OCT angiography could have been more appropriate to evaluate these cases . Ischemic change can at least in early phases cause swelling and axoplasmic flow statis thence increase rather than decrease in pRNFL and mGCC thickness

Visual field test mentioned in full details in Methodology , however no mention about the results of this test

Severity of the disease varied markedly between Prone Group and Non prone Group : statistical analysis should be employed to compensate for this difference

Cases with splinter hemorrhage and tortuous retinal veins can imply venous stasis or impending RVO , this can interfere with the results

IOP higher in Prone group related to the prone position , not explanation in discussion why this persists 6 months after recovery

Discussion requires revision ( Reviewer 2 )
