# Supplementary material for: The prone position in COVID-19 impacts the thickness of peripapillary retinal nerve fiber layers and macular ganglion cell layers
Source: PLoS One. 2024 May 2;19(5):e0300621. doi: 10.1371/journal.pone.0300621 (PMC11065264; doi:10.1371/journal.pone.0300621)
Supplement: Attachment — Submitted filename: Response to reviewers.docx [file pone.0300621.s006.docx]

**Response to reviewers**

**Dear Sir,**

Thank you very much for your constructive comments. Your feedbacks have been helpful in improving the quality of our manuscript. Here are the revisions.

**Reviewer 1**

The manuscript is well written and the interpreation of the findings sound. I just have 2 comments

1- The manuscript would have benefited from performing OCT angiography to study the ischemic changes if present. This could be added to the limitations.

**It was added to the limitations.**

**Page 22, line 393 :** Additionally, we suggest employing OCT angiography for further studies, as it is more suitable for evaluating ischemic changes.

2- I cant find the results of the visual field testing.

**It was added to the result section and S4 table.**

**Page 14, line 245 :** When the visual field parameters (visual field index, mean deviation, and pattern standard deviation) of the Prone and Non-Prone Groups at different time points were compared, no significant differences were observed. **S4 Table**

**Reviewer 2**

The research question is discussing a very novel point

The methodology and data analysis are presented in a very good manner

1. The discussion section requires revision and editing for data to be presented in a more organized and explanatory style

**Yes we did. The discussion underwent an overall revision, with a reorganization of its contents while retaining its original substance. Due to the diverse findings and aspects of this research, simplifying the organization was so difficult.**

**Editor**

1. Since Ischemic Optic Neuropathy is the main presumed pathogenesis , OCT angiography could have been more appropriate to evaluate these cases . Ischemic change can at least in early phases cause swelling and axoplasmic flow statis thence increase rather than decrease in pRNFL and mGCC thickness

**It was added to the limitations.**

2. Visual field test mentioned in full details in Methodology , however no mention about the results of this test

**It was added to the result section and S4 table.**

3. Severity of the disease varied markedly between Prone Group and Non prone Group : statistical analysis should be employed to compensate for this difference

**It was discussed in the discussion section (limitation)**

**Page 21, line 388 :** Furthermore, the severity of the disease was markedly higher in the Prone Group, primarily due to the natural characteristic of individuals who had to assume the prone position, potentially affecting neuroretinal changes. Although statistical analysis did not account for this difference, this study represents the first pilot investigation into this issue. Hence, a novel research endeavor with a more concise methodology is warranted.

4. Cases with splinter hemorrhage and tortuous retinal veins can imply venous stasis or impending RVO , this can interfere with the results

**We added your idea in** **the discussion section**

**Page 20, line 370 :** Moreover, retinal hemorrhages, particularly splinter hemorrhages, and tortuous retinal veins can be detected in cases of venous stasis resulting from prone positioning. It is important to note that these ocular manifestations are not attributed to COVID-19 infection.

5. IOP higher in Prone group related to the prone position , not explanation in discussion why this persists 6 months after recovery

**It was discussed in the discussion section**

**Page 16, line 275 :** The sustained elevation of intraocular pressure (IOP) observed in the Prone group is hypothesized to stem from prolonged prone positioning. This positioning may induce venous stasis and permanent venous dilatation, subsequently augmenting episcleral venous pressure and resulting in sustained elevated IOP levels.

6. Discussion requires revision ( Reviewer 2 )

**Yes we did.**

**Journal Requirements:**

1. Please ensure that your manuscript meets PLOS ONE's style requirements, including those for file naming. The PLOS ONE style templates can be found at

**We did it.**

2. We note that the grant information you provided in the ‘Funding Information’ and ‘Financial Disclosure’ sections do not match. When you resubmit, please ensure that you provide the correct grant numbers for the awards you received for your study in the ‘Funding Information’ section.

**We did it.**

3. Thank you for stating the following financial disclosure:

"Siriraj Research Fund, Faculty of Medicine, Siriraj Hospital, Mahidol University (grant number IO–R016531014.)"

Please state what role the funders took in the study. If the funders had no role, please state: ""The funders had no role in study design, data collection and analysis, decision to publish, or preparation of the manuscript.""

If this statement is not correct you must amend it as needed.

Please include this amended Role of Funder statement in your cover letter; we will change the online submission form on your behalf.

**It was added to the new cover letter.** "The funders had no role in study design, data collection and analysis, decision to publish, or preparation of the manuscript."

4. Please include your full ethics statement in the ‘Methods’ section of your manuscript file. In your statement, please include the full name of the IRB or ethics committee who approved or waived your study, as well as whether or not you obtained informed written or verbal consent. If consent was waived for your study, please include this information in your statement as well.

**It was added to the methods section Page 5, line 81 :** the Siriraj Institutional Review Board. All patients gave their written informed consent to participate

**5. We used PACE helps ensure that figures meet PLOS requirements then two new figures were uploaded.**
